# Does surgeon experience affect patient-reported outcomes 1 year after primary total hip arthroplasty? A register-based study of 6,713 cases in western Sweden

**DOI:** 10.1080/17453674.2018.1444300

**Published:** 2018-03-06

**Authors:** Per Jolbäck, Ola Rolfson, Maziar Mohaddes, Szilárd Nemes, Johan Kärrholm, Göran Garellick, Hans Lindahl

**Affiliations:** 1 Swedish Hip Arthroplasty Register, Gothenburg; 2 Department of Orthopaedics, Institute of Clinical Sciences, The Sahlgrenska Academy, University of Gothenburg, Gothenburg; 3 Department of Orthopaedics, Skaraborgs Hospital, Lidköping, Sweden

## Abstract

**Background and purpose:**

Several studies have reported on the influence of various factors on patient-reported outcomes (PROs) after total hip arthroplasty (THA), but very few have focused on the experience of the surgeon. We investigated any association between surgeons’ experience and PROs 1 year after primary THA.

**Patients and methods:**

Patient characteristics and surgical data at 10 hospitals in western Sweden were linked with PROs (EQ-5D-3L, Satisfaction Visual Analogue Scale (VAS), Pain VAS). These data were retrieved from the Swedish Hip Arthroplasty Register (SHAR). The surgeon’s level of experience was divided into 4 subgroups related to experience: < 8 years, 8–15 years, and >15 years of clinical practice after specialist certificate. If no specialist certificate was obtained the surgery was classified as a trainee surgery. Surgeons with >15 years’ experience as an orthopedic specialist were used as reference group in the analyses.

**Results:**

8,158 primary THAs due to osteoarthritis were identified. We identified the surgeons’ level of experience in 8,116 THAs. Data from SHAR on pre- and postoperative PROs and satisfaction at 1 year were available for 6,713 THAs. We observed a statistically significant difference among the 4 groups of surgeons regarding mean patient age, ASA classification, Charnley classification, diagnosis, and fixation technique. At 1-year follow-up, there were no statistically significant differences in Pain VAS, EQ-5D index, or EQ VAS among the subgroups of orthopedic specialists. Patients operated on by orthopedic trainees reported less satisfaction with the result of the surgery compared with the reference group.

**Interpretation:**

These findings indicate that patients can expect similar health improvements, pain reduction, and satisfaction 1 year after a primary THA operation irrespective of years in practice after specialty certification as an orthopedic surgeon.

During 2014, 16,565 total hip arthroplasties (THA) were carried out in Sweden, which corresponds to 331 procedures per 100,000 inhabitants aged 40 years or older (Garellick et al. 2014). The forecast increase in primary THAs in Sweden during the next 10–15 years is an annual volume of 18,000 in 2020 and 20,000 in 2030 (Nemes et al. 2014). During the last decade, there has been an increased focus on patient-reported outcomes (PROs) following primary THAs. One explanation of the increased interest in PROs might be a non-negligible group of patients reporting dissatisfaction after THA. Some studies report that 7–10% (Anakwe et al. 2011, Rolfson et al. 2011) of patients are uncertain about or dissatisfied with the result after THA.

A number of studies have investigated different factors influencing PROs after THA. In addition to demographic factors and socioeconomics, patient expectations and surgical factors have also been shown to influence PROs (Rolfson et al. 2011, Neuburger et al. 2013, Gordon et al. 2014, Greene et al. 2014, Jameson et al. 2014, Lindgren et al. 2014, Palazzo et al. 1999, Graves et al. 2016).

Traditionally in Sweden, training surgeons begin their education by assisting an experienced surgeon. After having performed a number of “more or less” supervised procedures followed by a postoperative discussion with their mentor, they perform the entire operation independently. After minimum of 5.5 years of training and achievement of the aims outlined for becoming an orthopedic specialist, trainees can apply for specialist certification at the Swedish National Board of Health and Welfare. The overall objective of the orthopedic education is that this learning procedure will not interfere with the outcome. In other surgical specialties, no statistically significant difference in outcomes after surgery have been shown between surgeons with different levels of experience (Bradbury et al. 1997, Naylor et al. 1998, Paisley et al. 1999, Goodwin et al. 2001, Asimakopoulos et al. 2006, Borowski et al. 2008). Nonetheless, surgical training programs may raise ethical issues. Patients might have a desire to be operated by a surgeon with long experience and this understandable desire might be in conflict with the education of surgeons. Only 3 studies have reported an association between surgeons’ experience and PROs after THAs. In a study based on data from the New Zealand Joint Registry (Inglis et al. 2013), surgeons had better results than trainees, whereas the other 2 found no difference (Palan et al. 2009, Reidy et al. 2016). In these 3 papers, there was a discrepancy in the classification of experience (i.e., consultant vs. non-consultant, consultant vs. supervised trainees, and unsupervised trainees and consultant vs. junior trainees and senior trainees) but also in which PROM (patient-reported outcome measures) instruments were used, which varied between Harris Hip Score (HHS) and Oxford Hip Score (OHS).

We were unable to find any study in which the experience of the surgeon was measured as the time after obtaining a specialist certificate in orthopedics and its relationship to PROs after THA.

Our main objective was to study any association between surgical experience and PROs 1 year after primary THAs. We hypothesized that patients operated on by more experienced orthopedic surgeons report better PROs when compared with patients operated on by less experienced surgeons.

## Patients and methods

The inclusion criteria were: a patient operated with a primary THA with either cemented, uncemented, hybrid, or reversed hybrid fixation and diagnostic indication of osteoarthritis (OA) as defined by International Statistical Classification of Diseases and Related Health Problems 10th Revision codes (ICD-10 codes) M16.0–M16.7 and M16.9. The surgery should also have been performed in a public hospital managed by the county council in the region of western Sweden between years 2007 and 2012. There should also be a complete preoperative and 1-year follow-up PROMs questionnaire, plus complete information on surgeon’s year for license to practice and/or specialist certification in orthopedics (Figure).

### Western Sweden

In 2012, the population in western Sweden amounted to around 1.6 million (17% of the total Swedish population). During the study period, 10 hospitals managed by the county council were performing primary THAs. In Sweden, the vast majority of hospitals are managed by the relevant county council.

### The Swedish Hip Arthroplasty Register (SHAR) PROMs program

The SHAR aims (Kärrholm 2010) to register all primary THAs and reoperations performed in Sweden. Participation is voluntary for both clinics and patients. The coverage is 100% (Garellick et al. 2012) for clinics and the completeness of individual patients is 98% (Garellick et al. 2014). Individual patient data, such as age, sex, height, weight, diagnosis, fixation technique, surgical approach, and type of implant used, as well as PRO data, were registered for every THA included in this study.

**Figure:**
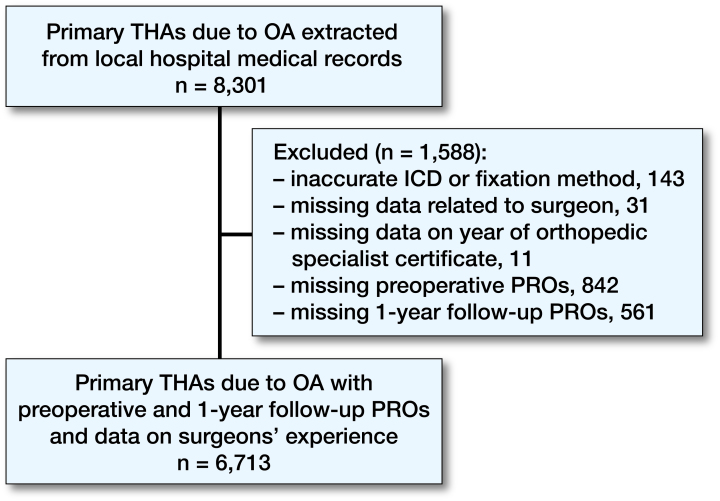
Flow chart.

We used data from the register’s PROM program which begun in 2002. The PROMs data were collected at the outpatient clinic visit shortly before surgery and at the 1-year follow-up by a mailed questionnaire. Non-respondents receive the first and only reminder after 8 weeks. The respondent rate for individual registration at national level in 2008 was 86% preoperatively and for both surveys 79% (Rolfson et al. 2011).

All the hospitals in western Sweden have been participating in collecting PROs since the start of the PROMs program.

The SHAR has used the 3-level version of EQ5D (EQ-5D-3L), which was developed by the EuroQol Group and introduced in 1990 (EuroQol 2015, Rabin and de Charro 2001). The EQ-5D index extends from minimum values of –0.594 (worse than death) to the maximum of 1.0 (full health) using a UK value set (Dolan 1997), as there is no specific Swedish value for the investigated period. The EQ-5D-3L also contains a health state visual analog scale (VAS) (EQ VAS) ranging from 0 (worst imaginable) to 100 (best imaginable). The PROMs program in the SHAR also includes questions on hip pain and satisfaction with the outcome of the operation. For the registration of pain, a VAS with a range of 0 (no pain) to 100 (worst pain imaginable) is used (Pain VAS). The question addresses the average pain experienced from the current hip during the last month. VAS is also used to address satisfaction with the outcomes of the surgery (hereafter only named “satisfaction”) with a range from 0 (satisfied) to 100 (dissatisfied) (Rolfson et al. 2011, Garellick et al. 2012, Kärrholm et al. 2015). In the SHARs PROMs program, there is also a question about musculoskeletal comorbidity based on the Charnley classification (Charnley 1972). This patient-reported Charnley classification is assigned by using 2 self-administered questions: “Do you have any symptoms from the other hip” and “Do you have problems walking because of other reasons” (e.g., pain from other joints, back pain, angina, or any other medical condition).

The difference between preoperative and the 1-year follow-up value was calculated for EQ-5D index, EQ VAS, and Pain VAS (Change Score). The Change Score could be interpreted as the amount of gain in quality of life and reduction of pain experienced by the patient 1 year after the operation.

### Sources of data

Local hospital medical records, the SHAR, and the Swedish National Board of Health and Welfare register of licensed health-care professionals were used as data sources. Data from local hospital medical records containing a 10-digit personal identity number, the name of the hospital, the name of the surgeon, the date of surgery, diagnosis, the fixation technique, and the ASA classification were extracted. This data were then linked with EQ-5D-3L, Charnley classification, Pain VAS, and Satisfaction VAS at 1-year follow-up from the SHAR. Patient characteristics, BMI, ASA classification, sex and age, diagnosis, and fixation technique were also available from the SHAR. Linking was performed using the 10-digit personal identity number, the name of the hospital, and the date of surgery.

For each surgeon involved, data on the year for license to practice and/or specialist certificate in orthopedics were obtained from publicly available data from the Swedish National Board of Health and Welfare register of licensed health-care professionals.

### Surgeons’ experience

Our classification of surgical experience was based on years between time for certification as an orthopedic specialist (both license to practice and orthopedic specialist certification) and the time of the THA surgery. If the surgeon has only a license to practice but no orthopedic specialist certification he was classified as an orthopedic trainee. Surgeons who did not appear with an orthopedic specialist’s certificate in the Swedish National Board of Health and Welfare register of licensed health-care professionals were classified as orthopedic trainees.

The surgeons were divided into 4 groups: orthopedic trainees, orthopedic specialists with less than 8 years’ experience, 8–15 years’ experience, and more than 15 years’ experience after specialist certification. The categorization of experience was decided in advance in the research group during the planning of the study.

### Statistics

SPSS version 21 (IBM Corp, Armonk, NY, USA) and R version 3.2.3 (R Foundation for Statistical Computing, Vienna, Austria) were used for the statistical analysis. We used the Kruskal–Wallis H test (variables: age, BMI, ASA classification, Charnley classification, fixation technique, diagnosis, EQ-5D index, EQ VAS, Pain VAS, Satisfaction VAS, EQ-5D index Change Score, EQ VAS Change Score, and Pain VAS Change Score) and Pearson’s chi-square test (gender). A linear regression analysis for EQ-5D index, EQ VAS, Pain Vas, and Satisfaction VAS (simple and multivariable) was performed. The regression coefficients (β) denote the change in the outcome for one-unit change in the exposure, or in case of categorical variables the mean difference between the reference and the different categories. Data were adjusted for age, sex, BMI, ASA classification, diagnosis, and Charnley classification at 1-year postoperatively. Surgeons with more than 15 years’ experience were used as a reference in the linear regression. The p-value for statistical significance was set at p < 0.05. Data in the linear regression are presented with a 95% confidence interval (CI).

### Sensitivity analysis

We performed 3 different sensitivity analyses with use of the same statistical methods as presented above except that other time limits for categorizing surgical experience were used. In first analysis surgical experience was categorized into 3 groups: trainees, 9 years or less, and 10 years or more as orthopedic specialist. In the second analysis there were 5 groups: trainees, 5 years or less, 6–9, 10–15, and 16 years or more as specialist. In the third analysis there were 4 groups now separated into trainees, 6 years or less, 7–18, and 19 years or more.

### Analysis of respondents versus non-respondents

A respondents versus non-respondents analysis was performed to evaluate whether non-responders differed in characteristic variables (age, sex, ASA classification, and BMI) and surgical data (fixation technique and diagnosis).

### Ethics, funding, and potential conflicts of interests

The study was approved by the Regional Ethical Review Board in Gothenburg, DNR 205-16. A research grant for the project was received from Skaraborgs Hospital research foundation. There is no conflict of interest.

## Results

The analyzed cohort comprised 6,713 primary THAs, of which 16% were performed at the university hospital, 29% at central hospitals and 56% at rural hospitals. A total of 219 surgeons performed the 6,713 cases. Of these cases, 8% were performed by trainees and 92% by orthopedic specialists.

### Patient characteristic

The sex distribution was even between the groups of surgeons. Less experienced surgeons operated on older patients than those who had longer experience (p < 0.001). There was a statistically significant difference in the distribution of ASA classification (p < 0.001) and Charnley classification (p < 0.001) between the groups of surgeons with different levels of experience. There was no statistically significant difference in BMI (p = 0.6). Patients with primary OA were more commonly operated on by less experienced surgeons (p < 0.001) (Table 1).

**Table 1.** Patient characteristics and surgical data divided into subgroups based on surgeons’ experience at the time of surgery

	Trainees (n = 538)	< 8 years (n = 2,181)	8–15 years (n = 984)	> 15 years (n = 3,010)	All (n = 6,713)	p-value
Age, years, mean						
All (SD)	73 (8)	71 (9)	69 (10)	67 (11)	69 (10)	< 0.001
Missing, n (%)	0 (0)	0 (0)	0 (0)	0 (0)	0 (0)	
Gender, n (%)						0.6
Male	215 (40)	935 (43)	424 (43)	1,264 (42)	2,838 (42)	
Missing, n (%)	0 (0)	0 (0)	0 (0)	0 (0)	0 (0)	
BMI, mean						0.2
All (SD)	27 (4)	28 (5)	27 (4)	27 (5)	27 (5)	
Missing, n (%)	150 (28)	527 (24)	287 (29)	854 (28)	1,818 (27)	
ASA classification, n (%)						< 0.001
I	91 (17)	580 (27)	326 (33)	882 (29)	1,879 (28)	
II	320 (60)	1,197 (55)	510 (52)	1,523 (51)	3,550 (53)	
III/IV	85 (16)	279 (13)	91 (9)	258 (9)	713 (11)	
Missing	42 (8)	125 (6)	57 (6)	347 (12)	571 (9)	
Diagnosis, n (%)						< 0.001
Primary OA	536 (100)	2,136 (98)	964 (98)	2,893 (96)	6,529 (97)	
Secondary OA	2 (0)	45 (2)	20 (2)	116 (4)	183 (3)	
Missing	0 (0)	0 (0)	0 (0)	1 (0.0)	1 (0)	
Fixation technique, n (%)						< 0.001
Cemented	481 (90)	1,749 (80)	764 (78)	2,034 (68)	5,028 (75)	
Uncemented	29 (5)	237 (11)	126 (13)	567 (19)	959 (14)	
Hybrids	5 (1)	62 (3)	21 (2)	82 (3)	170 (3)	
Reverse hybrids	23 (4)	133 (6)	72 (7.3)	322 (11)	550 (8)	
Missing	0 (0)	0 (0)	1 (0)	5 (0)	6 (0)	
Charnley classification at 1 year, n (%)						< 0.001
A	229 (43)	925 (42)	464 (47)	1,406 (47)	3,024 (45)	
B	52 (10)	179 (8)	87 (9)	307 (10)	625 (9)	
C	257 (48)	1,077 (49)	433 (44)	1,297 (43)	3,064 (46)	
Missing	0 (0)	0 (0)	0 (0)	0 (0)	0 (0)	

Kruskal–Wallis H test for age, BMI, ASA classification, diagnosis, fixation technique, and Charnley classification at 1-year follow-up, and Pearson’s chi-square test for sex. SD = standard deviation. BMI = body mass index. OA = osteoarthritis. ASA = American Society of Anesthesiologists.

### Surgical data

Cemented fixation had been used in 75%, uncemented in 14%, reverse hybrid in 8%, and hybrid in 3% of the hips. Trainees had a higher percentage of cemented THAs compared with more experienced surgeons. This percentage decreased as the experience of the surgeon increased in favor of an increasing proportion of hybrids and uncemented fixations (p < 0.001).

### Respondents versus non-respondents

Non-respondents rate in the PRO survey in our data totaled around 11% preoperatively and, pooled at 1-year follow-up, about 18%. There were some differences in patient demographics between respondents and non-respondents in the SHAR’s PROMs program. The non-respondents group were older (mean age 71) compared with the respondents (mean age 69) (p = 0.001). The sex distribution among respondents and non-respondents also differed, with a lower quota of males (39%) in the non-respondents group and 42% males among respondents. There was a higher proportion of ASA classification III/IV among the non-respondents (18% versus 11%, p < 0.001). Cemented fixation was also more common among non-respondents, 75% versus 62% among respondents (p = 0.01), but there were no differences regarding the diagnosis for the operation, with 96%/97% hips with primary OA in the non-respondents/respondents’ groups (p = 0.2). BMI was similar between responders (mean 27.5) and non-responders (mean 27.4).

### Outcomes

The preoperative EQ-5D index was lowest in the patient group operated on by the surgeons with the shortest time in practice and it increased in groups with increasing experience but with no statistically significant difference. At 1 year the values for EQ-5D index, EQ VAS, Pain VAS, and Satisfaction VAS were unevenly distributed among the groups with a tendency toward better results for those surgeons with longer experience but with no statistically significant difference (Table 2). The change scores from the preoperative evaluation to the follow up at 1 year, however, did not differ.

**Table 2.** PROs divided into subgroups based on surgeons’ experience at the time of surgery

	Trainees	< 8 years	8–15 years	> 15 years	All	p-value
EQ-5D index						
preoperative	0.41 (0.31)	0.41 (0.31)	0.41 (0.31)	0.43 (0.31)	0.42 (0.31)	0.05
1-year postop.	0.75 (0.24)	0.76 (0.25)	0.77 (0.25)	0.77 (0.24)	0.77 (0.24)	0.005
change	0.34 (0.35)	0.35 (0.34)	0.36 (0.34)	0.34 (0.34)	0.35 (0.34)	0.7
EQ VAS						
preoperative	56 (22)	56 (21)	57 (21)	57 (21)	57 (21)	0.2
1-year postop.	74 (20)	74 (21)	75 (20)	76 (20)	75 (20)	0.02
change	18 (23)	18 (23)	18 (22)	19 (23)	18 (23)	0.9
Pain VAS						
preoperative	61 (16)	61 (16)	61 (16)	60 (17)	61 (16)	0.6
1-year postop.	17 (19)	15 (19)	14 (18)	15 (18)	15 (18)	0.002
change	44 (23)	46 (23)	47 (22)	45 (23)	46 (23)	0.3
Satisfaction VAS						
1-year postop.	21 (23)	19 (22)	18 (21)	18 (22)	19 (22)	< 0.001

Kruskal–Wallis H test used for all variables. All values are presented as mean value and standard deviation (SD). VAS = visual analogue scale.

Patients operated on by surgeons with the shortest time in practice (both trainees and surgeons with less than 8 years’ experience) reported a higher value on the VAS for satisfaction (i.e., they were less satisfied) than those patients operated on by the most experienced surgeons (p < 0.001) (Table 2).

Both the simple and multivariable linear regression showed that longer time in practice as an orthopedic surgeon was not associated with better PROs 1 year after THAs measured by the EQ-5D index, EQ VAS. In terms of satisfaction and pain, the simple analysis showed that both trainees and the group with less than 8 years’ experience had a lower level of satisfaction and higher pain level compared with the most experienced surgeon with more than 15 years in orthopedics. However, after adjusting for differences in age, sex, BMI, diagnosis, ASA classification, and Charnley classification in the multivariable linear regression, there were no statistically significant differences in satisfaction at 1-year follow-ups for surgeons with less than 8 years’ experience. However, patients operated on by trainees reported lower satisfaction scores (Table 3).

**Table 3.** Linear regression analysis of PROs

	Simple sz-coefficient (95% CI)	Multivariable^a^ sz-coefficient (95% CI)
EQ-5D index		
15 years	Reference	Reference
8–15 years	0.00 (–0.02 to 0.02)	–0.01 (–0.03 to 0.01)
8 years	–0.01 (–0.03 to 0.00)	–0.01 (–0.03 to 0.01)
trainee	–0.02 (–0.05 to 0.00)	–0.02 (–0.05 to 0.00)
EQ VAS		
15 years	Reference	Reference
8–15 years	–0.05 (–1.50 to 1.41)	–0.82 (–2.47 to 0.83)
8 years	–1.29 (–2.40 to –0.17)	–0.45 (–1.71 to 0.80)
trainee	–1.79 (–3.65 to 0.07)	–0.91 (–3.03 to 1.21)
Pain VAS		
15 years	Reference	Reference
8–15 years	–0.14 (–1.46 to 1.18)	0.49 (–1.06 to 2.05)
8 years	0.84 (–0.17 to 1.85)	0.78 (–0.41 to 1.96)
trainee	2.37 (0.69 to 4.05)	1.88 (–0.12 to 3.88)
Satisfaction VAS		
15 years	Reference	Reference
8–15 years	–0.22 (–1.81 to 1.37)	0.55 (–1.32 to 2.42)
8 years	1.58 (0.37 to 2.80)	0.72 (–0.70 to 2.13)
trainee	3.61 (1.58 to 5.63)	2.64 (0.24 to 5.03)

a Adjusted for age, sex, BMI, ASA classification, diagnosis, and Charnley classification at 1 year. CI = confidence intervals, BMI = body mass index, ASA = American Society of Anesthesiologists, VAS = visual analogue scale. Reference is surgeon with >15 years’ experience as orthopedic specialists.

All 3 sensitivity analyses showed similar results with respect to the distribution of patient characteristics, surgical data, and PROs (data not shown).

## Discussion

Our main objective was to study any association between surgical experience and PROs 1 year after primary THAs. We did not find any association between surgeons’ experience and EQ-5D index, EQ VAS, and Pain VAS 1 year after primary THA. However, patients operated on by orthopedic trainees reported lower satisfaction VAS at 1-year follow-up than those operated by surgeons with more than 15 years’ experience.

Our findings are in accordance with those reported by Palan et al. (2009) and Reidy et al. (2016), with similar PROs except for less satisfaction with the outcome of surgery in patients operated on by trainees. Inglis et al. (2013) recorded the OHS in the New Zealand Joint Registry at 6 months and related this outcome to surgeons’ experience. They found a higher mean OHS for patients operated on by surgeons compared with operations performed by supervised and unsupervised trainees. Their study included only 6-month follow-up data on 20% of randomly selected patients and no adjustment was made for preoperative PROs as these data not were collected. Thus, any potential difference in baseline values between the subgroups could not be accounted for.

In our study, there were significant differences in patient characteristics between those operated by more or less experienced surgeons and there was also a statistically significant difference between the groups of surgeons as regards choice of fixation technique. This bias in patient selection and fixation technique could be expected because, in Sweden, trainees usually start by learning cemented fixation, which is still the gold standard (Troelsen et al. 2013). Uncemented components are regarded as being more difficult to handle because of the risk for complications arises intraoperatively (Kärrholm et al. 2015) and this method is commonly used by surgeons later in their career. The reason for the difference in mean age between the groups could be an effect of selection of type of fixation. Uncemented, hybrid, and reverse hybrid are more suitable in younger patients. The difference in mean age between the subgroups may also affect the observed difference in ASA classification due to the fact that younger patients are expected to be healthier.

We did note the same differences in postoperative PROs as those reported by Inglis et al. (2013). However, after adjusting for patient characteristics and surgical data, these differences became statistically non-significant.

Non-respondent rate in the PRO survey in our data totaled around 11% preoperatively and, pooled at 1-year follow-up, about 18%. This is better than in the SHARs PROM for all clinics in Sweden where the preoperative respondent rate was 14% and in both surveys 21%. Females at a greater age, with higher ASA classification and treated with cemented fixation, were underrepresented among the respondents in our study. The reason for this is not known, but high age and presence of more concomitant diseases could have had an influence. This patient group could be expected to report poorer health status and quality of life, but whether excess dropout of these patients actually had any influence on the results remains unknown. One could speculate that the majority of these patients were operated by less experienced surgeons and thus, rather, would make the differences observed more obvious than vice versa. Overall the pooled response rate of 82% is probably difficult to improve in a study like this. Despite the skewed distribution in dropouts we think that the conclusions made in our study are reasonably valid.

Our study has limitations. The extent to which the experience of the surgeon restricted to the annual number of hip arthroplasties is reflected in patient-reported outcomes is unclear. The surgeon might have operated on an unknown number of fractures and undertaken other types of orthopedic surgery, for example. The comparatively wide variation among surgeons regarding the volume of primary THAs might partly be an effect of the performance of other types of arthroplasty, including hip and knee revisions. Some surgeons might therefore have a much larger total annual volume of operations, including hip revisions and hemiarthroplasties, which could be a source of bias when compared with surgeons with a low overall surgical volume. However, we decided to subgroup surgeons based on years after specialist degree rather than number of THR surgeries. In this this way experience from other type of operations than primary THR and clinical practice in general, which includes a continuing learning process, will be included.

We lack information on socioeconomic factors and educational level, which are known to influence PROs (Borowski et al. 2008, Neuburger et al. 2013). These factors are not available in SHAR or local medical records. In this study, we did not link our data with Statistics Sweden (Greene et al. 2014, Krupic et al. 2014). This lack of information may affect satisfaction if there is a skewed distribution of patients with a good economic situation and a high educational level in any of the groups.

We have no information on the quality of the preoperative information given to the patient, or whether this information was given only verbally, only in writing, or both. Nor do we know whether the preoperative information was given by the same surgeon who performed the operation, or whether the information was repeated. However, there is little evidence in the literature to support the value of preoperative patient education (McDonald et al. 2004, Aydin et al. 2015), which might suggest that its presence may have no or a minor influence on the outcome after surgery.

Another limitation is that we do not have any knowledge of the supervision of the trainee. The level of supervision may vary between completely unsupervised to fully supervised individuals. Earlier studies have, however, not shown any substantial difference in PROs after up to 10 years of follow-up between unsupervised and supervised trainees (Reidy et al. 2016). It is therefore also possible that this potential confounder had a limited influence in our study.

Of the possible confounders mentioned here, we feel that socioeconomic factors and continued patient treatment by one and the same surgeon may have the greatest influence on PROs and patient satisfaction. These hypotheses require further studies.

As highlighted above there are a number of confounding factors that we could not consider, and likely there is a degree of residual confounding that we could not control for. As with other observational studies, the results of this study should be considered in terms of association rather than causality.

In summary, to our knowledge, this is the largest report analyzing the influence of surgeon experience on postoperative PRO adjusted for Charnley classification and comorbidity. We found considerable differences in patient characteristics and surgical data depending on surgeons’ experience. After adjusting for these covariates, we found no associations between orthopedic trainees and/or orthopedic specialists with variable length of practice after certification and EQ-5D index, EQ VAS, and Pain VAS 1 year after primary THA. Patients operated on by orthopedic trainees did, however, report less satisfaction VAS with the outcome of surgery than those patients operated on by surgeons with more than 15 years’ practice. No such difference in level of satisfaction was observed between any of the orthopedic specialist subgroups. These findings indicate that patients can expect similar health improvements, pain reduction, and satisfaction 1 year after a primary THA operation irrespective of years in practice after specialty certification as orthopedic surgeon.

PJ had the original idea for the study, processed the data, undertook the writing of the manuscript, and performed the statistical analyses but not the linear regression analysis. SN performed the linear regression analysis. OR, MM, JK, GG, and HL took part in the interpretation of the data and in writing the manuscript. GG contributed significantly to the prolonged ethical approval process. All the authors have read and approved the final manuscript.

*Acta* thanks Yvette Pronk and other anonymous reviewers for help with peer review of this study.
